# Posttraumatic Stress Disorder and Depression Symptom Severities Are Differentially Associated With Hippocampal Subfield Volume Loss in Combat Veterans

**DOI:** 10.1177/2470547017744538

**Published:** 2017-12-13

**Authors:** Christopher L. Averill, Ritvij M. Satodiya, J. Cobb Scott, Kristen M. Wrocklage, Brian Schweinsburg, Lynnette A. Averill, Teddy J. Akiki, Timothy Amoroso, Steven M. Southwick, John H. Krystal, Chadi G. Abdallah

**Affiliations:** 1 130477 National Center for PTSD, Clinical Neurosciences Division, US Department of Veterans Affairs, West Haven, CT, USA; 2 Department of Psychiatry, 12228 Yale University School of Medicine, New Haven, CT, USA; 3 Department of Psychiatry, Perelman School of Medicine, University of Pennsylvania, Philadelphia, PA, USA; 4 VISN4 Mental Illness Research, Education, and Clinical Center, Philadelphia VA Medical Center, Philadelphia, PA, USA; 5 Gaylord Specialty Healthcare, Department of Psychology, Wallingford, CT, USA

**Keywords:** posttraumatic stress disorder, depression, hippocampus, hippocampal subfield, hippocampal volume, hippocampus–amygdala-transition-area, cornu ammonis, dentate gyrus, long axis, neuroimaging, Veteran, magnetic resonance imaging

## Abstract

**Background:**

Two decades of human neuroimaging research have associated volume reductions in the hippocampus with posttraumatic stress disorder. However, little is known about the distribution of volume loss across hippocampal subfields. Recent advances in neuroimaging methods have made it possible to accurately delineate 10 gray matter hippocampal subfields. Here, we apply a volumetric analysis of hippocampal subfields to data from a group of combat-exposed Veterans.

**Method:**

Veterans (total, n = 68, posttraumatic stress disorder, n = 36; combat control, n = 32) completed high-resolution structural magnetic resonance imaging. Based on previously validated methods, hippocampal subfield volume measurements were conducted using FreeSurfer 6.0. The Clinician-Administered PTSD Scale assessed posttraumatic stress disorder symptom severity; Beck Depression Inventory assessed depressive symptom severity. Controlling for age and intracranial volume, partial correlation analysis examined the relationship between hippocampal subfields and symptom severity. Correction for multiple comparisons was performed using false discovery rate. Gender, intelligence, combat severity, comorbid anxiety, alcohol/substance use disorder, and medication status were investigated as potential confounds.

**Results:**

In the whole sample, *total hippocampal volume* negatively correlated with Clinician-Administered PTSD Scale *and* Beck Depression Inventory scores. Of the 10 hippocampal subfields, Clinician-Administered PTSD Scale symptom severity negatively correlated with the *hippocampus–amygdala transition area* (HATA). Beck Depression Inventory scores negatively correlated with dentate gyrus, cornu ammonis 4 (CA4), HATA, CA2/3, molecular layer, and CA1. Follow-up analysis limited to the posttraumatic stress disorder group showed a negative correlation between Clinician-Administered PTSD Scale symptom severity and each of HATA, CA2/3, molecular layer, and CA4.

**Conclusion:**

This study provides the first evidence relating posttraumatic stress disorder and depression symptoms to abnormalities in the HATA, an anterior hippocampal region highly connected to prefrontal-amygdala circuitry. Notably, dentate gyrus abnormalities were associated with depression severity but not posttraumatic stress disorder symptoms. Future confirmatory studies should determine the extent to which dentate gyrus volume can differentiate between posttraumatic stress disorder- and depression-related pathophysiology.

## Introduction

Reductions in hippocampal volume have been associated with posttraumatic stress disorder (PTSD). Although not without inconsistencies,^1^ an initial description of hippocampal reduction in combat-related PTSD^2^ led to many magnetic resonance imaging (MRI) replications in civilians and Veterans.^1,3–6^ However, the studies of structural hippocampus alterations in PTSD predominantly considered the hippocampus complex as a single structure (i.e., volume of the entire right or left hippocampus)^7,8^ with little investigation of the relationship between psychopathology and subfields of the hippocampus.^7^ A growing body of clinical and preclinical literature has, however, suggested a number of possible models for specialization across the long axis of the hippocampus. Two recent papers provide an in-depth discussion and synthesis.^9,10^ Briefly, anterior and posterior aspects of the hippocampus may serve different functions in terms of pattern separation and pattern completion and do not share reciprocal connections with the same cortical or subcortical areas of the brain.^9^ Identification of specific subfield abnormalities in PTSD may have particular pathophysiological and treatment implications and add to our understanding of both the role of the hippocampal subfields and the way those roles may shape (or be shaped by) a specialization gradient along the hippocampus.

The hippocampus can be segmented into 10 gray matter subfields^7,11^ (described further in Table 1; see Video 1 for animated 3D visualization http://journals.sagepub.com/doi/suppl/10.1177/2470547017744538), including the cornu ammonis (CA) regions 1–4, subiculum (SUB), presubiculum (PrSUB), parasubiculum (PaSUB), granule cell and molecular layers (MLs) of the dentate gyrus (DG), hippocampal–amygdala transition area (HATA), ML of the hippocampus, and the hippocampal tail. These hippocampal subfields can be defined by their unique cellular architectures and distinct developmental and functional properties.

**Table 1.** Anatomy and putative function of the gray matter hippocampal subfields.

Subfield	Clinical and preclinical notes
HATA	Part of the anterior HPC, bounded by the HPC folds, lateral ventricle, and alveus.^11^ Carries afferent and efferent signals between the rest of the HPC and the caudal amygdala and may send axonal collaterals to the PFC and hypothalamus.^12^ Plausible role in acquiring traumatic memories, behavioral and neuroendocrine response to threat, fear conditioning,^13^ and consolidation of contextual fear learning and memory.^14^ Projections to the HTH and PFC may relate to aggressive or defensive sexual behavior.^12^
ML	Sits above the SUB directly beneath the HPC fissure; traces the HPC folds along the CA fields and SUB.^11^ Interneuronal synaptic connections, serving probable role of transmission and integration of information across the HPC.^15^ Volume reduction has been observed in BP.^16^
DG	Tri-layered structure beginning near the middle of the HPC head.^11^ Supports neurogenesis, memory formation, and neuroplasticity; rapid acquisition in spatial memory involving pattern separation (young granule cells) or pattern completion (mature granule cells).^17,18^ Volumetric atrophy may be associated with chronic stress,^19^ BD,^16^ depression,^20^ PTSD,^21,22^ and schizophrenia.^23,24^
CA4	Hilus of the DG.^11^ Receives excitatory inputs from the cerebral cortex and interneurons of the DG; contains mRNA related to neurogenesis, complexins which are important for neurotransmission,^25^ and BDNF which is important for survival and maturation of neurons.^26^ Various reductions in mRNA concentrations are associated with BD, possibly dysregulating neuronal transmission.^25^
CA2/3	Superior to DG from posterior half of HPC head to the HT.^11^ Extensive interconnections among principal cells forming an autoassociative network, supports formation of arbitrary spatial association, temporary maintenance of spatial working memory, and spatial pattern completion.^27–30^ Supports mnemonic processes in the formation of accurate spatial memory.^31^ Implicated in spatial pattern separation via interaction of mossy fibers/DG^29^ and acquisition of context-dependent fear extinction but not context-dependent fear memories.^32^ Influences discrete gene expression.^10^ Volume alterations found in PTSD.^21^
CA1	Extends from SUB, ending near first HPC fold.^11^ Trisynaptic loop created by CA1 projections through SUB to entorhinal cortex relates to acquisition of memory and spatial learning.^33^ Context-dependent fear extinction and retrieval of contextual memory.^24,32^ Insult to CA1 impairs spatial navigation and working memory, but not reference memory.^33,34^ Reduction in volume seen with untreated schizophrenia.^35^
SUB	Three-layered allocortex, lateral to PrSUB and CA1.^11^ Part of limbic memory system, responsible for mnemonic processing,^36^ short-term memory retrieval, and spatial encoding.^15^ Stress response and inhibitory control over the HPA^37^ and implications in fear conditioning.^38^ Modulates epileptic discharges from the HPC.^15^ Atrophy implicated in early and advanced AD.^39^
PrSUB	BA27; periallocortex; heavily myelinated ML; sits along HPC fissure, anterior to retrosplenial region.^11^ Connection with excitatory synapses supports bursting behaviors.^40^ Short-term memory and processing of spatial location information.^41^ Implicated in and possible marker for AD.^39^
PaSUB	BA49; periallocortex; smaller than PrSUB and SUB; sits along HPC fissure.^11^ Continuous recognition memory for spatial navigation^41,42^ and integration of head direction.^42^ Inputs from GABAergic medial septal neurons and expression of cholinergic activity markers.^43^ Projecting neurons in thalamus, SUB, and PrSUB.^44^ Aids in determining spike timing relative to theta oscillations.^45^
HT	Not well studied, so difficult to make reliable annotations. Volume reductions reported with increasing duration/severity of BD.^46^ FMRI evidence shows that HT may activate with abnormal ease in PTSD.^47^

HPC: hippocampus; HATA: hippocampal–amygdala transition area; ML: molecular layer of the HPC; DG: granule cell and ML of the dentate gyrus; CA: cornu ammonis; SUB: subiculum; PrSUB: presubiculum; PaSUB: parasubiculum; HT: HPC tail; AD: Alzheimer’s disease; BA: Broadmann area; BDNF: brain derived neurotrophic factor; BP: bipolar disorder; HPA: hypothalamus–pituitary–adrenal axis; HTH: Hypothalamus; PFC: prefrontal cortex.

Methods for delineating hippocampal subfields in human subjects have evolved.^7,8,48,49^ They are now included in software packages like FreeSurfer (https://surfer.nmr.mgh.harvard.edu). The use of different software packages, probabilistic atlases, or even hand-drawn segmentation of the hippocampus may explain some contradictory results reported in the literature.^7,48^ Recently, a new atlas^11^ derived from .13 mm resolution *ex vivo* MRI and 1 mm *in vivo* MRI was included in the release of FreeSurfer Version 6.0, providing more reliable and specific segmentation^11^ than was available previously. While preclinical evidence suggests hippocampal subfields are specialized in function,^7^ the historical challenge of *in vivo* segmentation has limited the exploration of subfields in humans, leading to volumetric investigations of less refined segmentation such as head, body, tail;^50–53^ or SUB, CA1-3, DG, and entorhinal cortex.^21,54–57^

Studies of hippocampal subfields in major depression and stress-related psychopathology found significant negative correlations between depression and DG,^58–60^ CA,^59,60^ and SUB,^60^ and between stress sequelae and CA1,^61^ CA3,^61–63^ and DG.^61–63^ However, less is known about hippocampal subfield abnormalities in PTSD. One previous study (17 PTSD; 19 non-PTSD) reported lower DG/CA3 volume in PTSD, but no correlation between hippocampal subfields and PTSD symptom severity.^21^ Secondary analysis in the combined sample found a negative correlation between DG/CA3 volume and insomnia severity.^57^ Although this pioneering pilot study had many strengths, it was also limited by its relatively small sample, with some participants unexposed to trauma, and the fact that their DG/CA3 region included the DG, CA3, CA4, and a large part of the ML. Another, more recent study reported a negative correlation between combined DG/CA4 subfield volume and PTSD symptom severity among 97 military Veterans.^22^ However, this study did not investigate a more comprehensive segmentation of the hippocampal subfields. To advance this line of research, we employed a *state-of-the-art* segmentation of 10 gray matter hippocampal subfields in a large sample of all combat-exposed US Veterans. In addition, to capture the association between a continuum of PTSD symptom severity and biological abnormalities,^64,65^ our primary analysis investigated the correlation between clinical severity and hippocampal subfields regardless of PTSD diagnosis.

Better understanding of the role that hippocampal subfields play in trauma- and stress-related psychopathology can provide further insight into the neural mechanisms underlying PTSD and depression and may prove to be relevant to drug development and treatment strategies. To our knowledge, the present study is among the first to evaluate the 10 gray matter hippocampal subfield volumes and is the first study to do so within the context of PTSD. Yet, considering prior literature,^21,58–63^ we predicted that the severity of both PTSD and depression symptoms would be associated with reduced volumes in hippocampal subfields.

## Methods

### Participants

This study includes 68 combat-exposed US Veterans between the age of 21 and 60 years who provided informed consent to participate in a study approved by the Human Studies Subcommittee (Institutional Review Board) at the VA Connecticut Healthcare System. The present sample has also been investigated in previous publications reporting cortical thickness reductions,^65^ morphometric abnormalities,^66^ and functional dysconnectivity^64^ associated with PTSD; however, the hippocampal subfields in this cohort are being investigated here for the first time. Participants were excluded if presenting with a psychotic disorder, bipolar depression, learning disorder or attention deficit disorder/attention deficit hyperactivity disorder, or moderate-to-severe traumatic brain injury. Epilepsy, brain tumors, and other gross neurological disorders with anatomical consequence were also excluded. Participants taking benzodiazepines or with standard MRI contraindications were not included in the study. Benzodiazepines were excluded because of their potential effects on the functional MRI scans, which were acquired as part of the parent study. In order to ensure generalizability of any findings, subjects with PTSD and highly co-occurring comorbidities such as unipolar depression, anxiety disorders, substance or alcohol use disorders, and those who were taking a stable dose of antidepressants were allowed to participate. These factors were evaluated as potential confounds in post hoc analyses but were largely not found to correlate with the outcomes presented below.

### Clinical Measurement

PTSD diagnosis and symptom severity were determined using the Clinician-Administered PTSD Scale (CAPS) for the DSM-IV.^67,68^ Depressive symptoms were evaluated with the Beck Depression Inventory (BDI) Second Edition.^69^ The Structured Clinical Interview for the DSM-IV^70^ was used to evaluate psychiatric comorbidities. Combat exposure was measured with the Combat Exposure Scale (CES).^71^ Premorbid IQ was estimated using the Wechsler Test of Adult Reading (WTAR).^72^

### Neuroimaging

Structural MRI (sMRI) acquisitions were conducted in a 3 Tesla magnetic field using a Siemens TIM Trio scanner and a 32-channel head coil. Following a three-plane localization, two MPRAGE scans providing T1-weighted contrast (TR = 2530 ms; TE = 2.71 ms; TI = 1200 ms; Flip = 7°) were acquired. All sMRIs were acquired with the same 256 mm FOV and 1 × 1 × 1 mm isotropic voxels. Volumetric hippocampal subfield estimates were conducted using the fully automated process provided by FreeSurfer^73^ Version 6.0 (https://surfer.nmr.mgh.harvard.edu) as described in previous studies^74–76^ and in the Supplementary Material. After completing the FreeSurfer “recon-all” pipeline, the following command was used to segment bilateral hippocampi and their 10 gray matter subfields (Code 1). Due to the lack of distinguishing contrast and the small size of the CA2 subfield, the CA2 and CA3 were combined, which are discussed below as the CA2/3.

# Code 1:

recon-all -s $subject –hippocampal-subfields-T1

Following data processing, data quality was assessed by checking recon-all segmentation quality and checking subfields and fissure images visually for discorrelation between segmentation and full region, outliers in each subfield after correcting for total intracranial volume (TIV), strong bilateral asymmetries, and unexpected subfield volumes. No manual intervention was necessary upon completion of quality evaluation, and these steps were undertaken while blinded to the demographic and clinical characteristics related to each scan.

### Statistical Analysis

Participant demographics and psychiatric variables are presented in Supplemental Table S1. To examine the relationship between the hippocampus and severity of either PTSD symptoms (primary analysis) or depressive symptoms (secondary analysis), two-tailed partial correlations of each hippocampal subfield and the specific dimensional outcome were conducted after testing for normality (and log transforming as required). Age and TIV were included as covariates in all analyses. We included TIV as a covariate in our analyses, consistent with much of the literature;^51–53,55,56,58–60,63^ however, other methods are also commonly employed to adjust each volume of interest based on TIV. The reader should be aware that the “residual” method^21,54^ may yield different results than the covariate method employed here. Additional discussion of TIV correction can be found in the literature.^77,78^ To investigate for potential confounds, we repeated the partial correlation analyses controlling for each of the following variables: gender, WTAR, CES, comorbid anxiety, alcohol/substance use disorder, and medication status. For the full sample primary analyses, correction for multiple comparisons was performed using false discovery rate.^79^ Our secondary analyses in PTSD-only or medication-free subgroups were conducted with exploratory intent to inform future research on any significant or trending areas of interest. For this reason, we did not correct these follow-up analyses for multiple comparisons. For additional information about the effects of age and PTSD severity, see the Supplementary Material.

## Results

### Primary Analysis: PTSD Severity and Hippocampal Subfields

**Figure 1.**
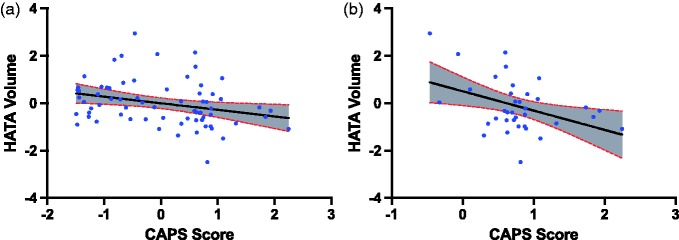
PTSD severity (CAPS score) is associated with HATA volume. (a) Primary analysis in full group. (b) Exploratory analysis in PTSD subgroup. CAPS score and HATA volume are residuals after controlling for age and total intracranial volume. CAPS: Clinician-Administered PTSD Scale.

In the full sample, CAPS symptom severity negatively correlated with total hippocampal volume (*r* = −0.32, *p* = 0.008, *df* = 64). Among the 10 hippocampal subfields, the HATA volume significantly correlated with CAPS severity (*r* = −0.34, *p_fdr_* = 0.05, *df* = 64; Figure 1, Table 2). This correlation remained significant after controlling for each of gender, intelligence, combat severity, comorbid anxiety, alcohol/substance use disorder, and medication status. CAPS score correlations with CA1 and CA2/3 showed statistically nonsignificant trend before correction for multiple comparisons. See “CAPS (full group)” columns in Table 2 for additional results. See Supplemental Table S4 for raw subfield volumes.

**Table 2.** Partial correlation of hippocampal subfields and PTSD severity.

Hippocampal subfield	CAPS (full group)				CAPS exploratory (PTSD group)		
	*r*	*p*	FDR	*df*	*r*	*p*	*df*
Total hippocampus	−.32	.01*	—	64	−.42	.02*	32
Parasubiculum	.11	.36	.41	64	−.16	.37	32
Presubiculum	.05	.67	.67	64	−.06	.73	32
Subiculum	−.11	.37	.41	64	−.23	.20	32
CA1	−.22	.08	.23	64	−.33	.06	32
CA2/3	−.22	.07	.23	64	−.40	.02*	32
CA4	−.17	.17	.28	64	−.40	.05*	32
Dentate gyrus	−.16	.20	.28	64	−.33	.06	32
HATA	−.34	.01*	.05*	64	−.41	.02*	32
ML of hippocampus	−.20	.11	.23	64	−.35	.04*	32
Hippocampal tail	−.20	.12	.23	64	−.21	.23	32

r: partial correlation controlling for age and total intracranial volume; FDR: significance after correction for multiple comparison; CA: cornu ammonis; ML: molecular layer; DG: granule cell and molecular layers of the dentate gyrus; HATA: hippocampal–amygdala transition area; CAPS: Clinician-Administered PTSD Scale for DSM-IV.

* *p* ≤ 0.05. Exploratory analysis did not correct for multiple comparisons.

To further characterize the primary findings, we conducted a follow-up analysis limited to the PTSD subjects. In this exploratory analysis, CAPS score was correlated with total hippocampal volume (*r* = −0.42, *p* = 0.02, *df* = 32), CA2/3 volume (*r* = −0.40, *p* = 0.02, *df* = 32), CA4 (*r* = −0.34, *p* = 0.05, *df* = 32), HATA (*r* = −0.41, *p* = 0.02, *df* = 32), and ML (*r* = −0.35, *p* = 0.04, *df* = 32). No significant correlations were observed between CAPS symptom severity and the other gray matter subfields. See “CAPS Exploratory (PTSD group)” columns in Table 2 for additional results.

### Secondary Analysis: Depression Severity and Hippocampal Subfields

**Figure 2.**
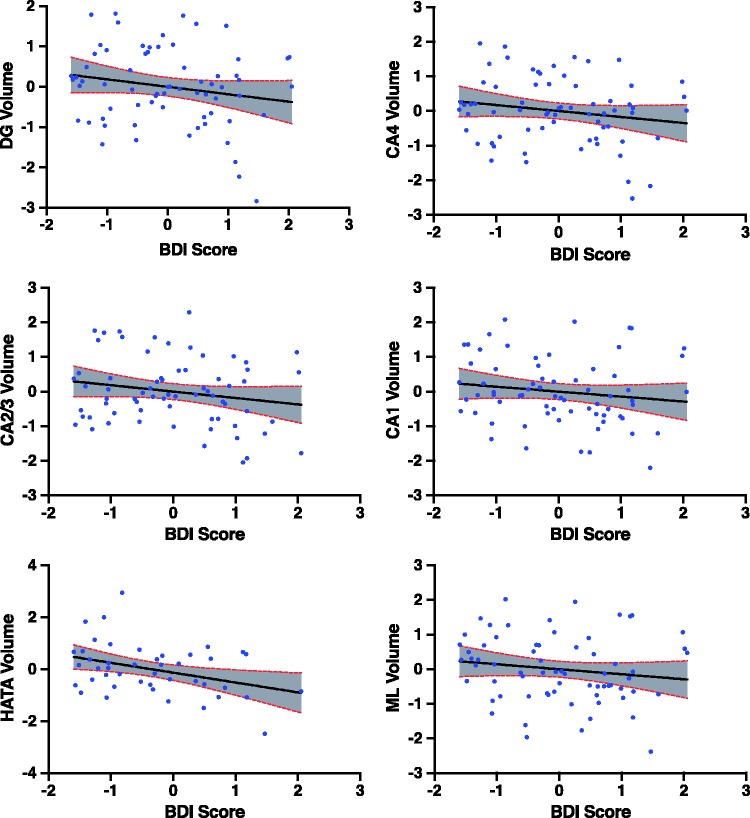
Depression severity (BDI score) is associated with multiple hippocampal subfield volumes. BDI score and subfield volumes in the full group analysis are residuals after controlling for age and total intracranial volume. BDI: Beck Depression Inventory; CA: cornu ammonis; ML: molecular layer.

In the full sample (Table 3), BDI score negatively correlated with total hippocampal volume (*r* = −0.32, *p* = 0.01, *df* = 64). Among the 10 hippocampal subfields (Figure 2, Table 3), BDI negatively correlated with the DG (*r* = −0.33, *p_fdr_* = 0.04, *df* = 64), HATA (*r* = −0.30, *p_fdr_* = 0.04, *df* = 64), CA1 (*r* = −0.27, *p_fdr_* = 0.05, *df* = 64), CA2/3 (*r* = −0.30, *p_fdr_* = 0.04, *df* = 64), CA4 (*r* = −0.32, *p_fdr_* = 0.04, *df* = 64), and the ML of the hippocampus (*r* = −0.29, *p_fdr_* = 0.04, *df* = 64). These correlations remained significant after controlling for each of gender, intelligence, combat severity, comorbid anxiety, and alcohol/substance use disorder. Controlling for medication status, only the correlation between BDI and HATA remained significant. Thus, to further explore the putative confounding effects of medications, we performed a follow-up analysis in which the partial correlations between BDI and hippocampal subfields were repeated in a restricted sample including only those participants who were medication-free (n = 44). In this unmedicated subgroup, BDI showed a comparable pattern of negative correlation coefficient, but only the correlation with total hippocampal volume (*r* = −0.30, *p* = 0.05, *df* = 40) and HATA (*r* = −0.37, *p* = 0.02, *df* = 40) were statistically significant in this smaller sample. Nonsignificant trends were seen with CA1 and CA2/3. See “BDI Exploratory (med-free only)” columns in Table 3 for additional results.

**Table 3.** Partial correlation of hippocampal subfields and depression severity.

Hippocampal subfield	BDI (full group)				BDI exploratory (med-free only)		
	*r*	*p*	FDR	*df*	*r*	*p*	*df*
Total hippocampus	−.32	.01*	—	64	−.30	.05*	40
Parasubiculum	.05	.70	.78	64	.06	.69	40
Presubiculum	−.04	.78	.78	64	.07	.67	40
Subiculum	−.12	.36	.45	64	−.01	.97	40
CA1	−.27	.03*	.05*	64	−.27	.09	40
CA2/3	−.30	.02*	.04*	64	−.28	.07	40
CA4	−.32	.01*	.04*	64	−.21	.19	40
Dentate gyrus	−.33	.01*	.04*	64	−.25	.11	40
HATA	−.30	.02*	.04*	64	−.37	.02*	40
ML of hippocampus	−.29	.02*	.04*	64	−.26	.10	40
Hippocampal tail	−.13	.29	.41	64	−.07	.65	40

r: partial correlation controlling for age and total intracranial volume; FDR: significance after correction for multiple comparison; CA: cornu ammonis; ML: molecular layer; DG: granule cell and MLs of the dentate gyrus; HATA: hippocampal–amygdala transition area; BDI: Beck Depression Inventory, Second Edition.

* *p* ≤ 0.05. Exploratory analysis did not correct for multiple comparisons.

## Discussion

Although not without inconsistencies, replicated evidence supports the presence of volumetric alterations in the hippocampus among those with PTSD.^1,3,4,6,51,53,80–83^ The current study results provide further evidence of selective volumetric variability within the hippocampal subfields related to PTSD and depressive symptoms. The severity of PTSD and depression symptoms were associated with reduced volumes in certain hippocampal subfields. Our primary analysis revealed a novel finding of negative association between PTSD symptom severity and the volume of the HATA subfield, an area critically positioned between the amygdala and anterior hippocampus. In the full sample, PTSD severity did not correlate with either the DG or the CA2/3, subfields previous implicated in PTSD pathophysiology.^21^ However, a follow-up exploratory analysis showed a negative correlation between symptom severity and CA2/3 in Veterans meeting diagnostic criteria for PTSD. The ML and CA4 subfields were also negatively associated with clinical severity within the PTSD group. Consistent with the hypothesized role of the DG in depression pathology,^84^ our findings confirmed a negative correlation between depression symptom severity and volume of the DG subfield. HATA and other core hippocampal subfields (CA1, CA2/3, CA4, and ML) were also negatively associated with depression severity. However, our data could not rule out the possibility that the correlations between depression severity and hippocampal subfields are confounded by medication status, considering the failure to maintain statistical significance in the medication-free subgroup—although this could be due to the lack of statistical power in a relatively smaller sample.

In both primary and secondary analyses, the HATA volume was significantly associated with symptoms severity. Specifically, HATA volume was negatively correlated (and to nearly the same magnitude) with severity of both PTSD and depressive symptoms, regardless of medication or diagnostic status. The HATA is directly connected to the amygdala and also communicates with the prefrontal cortex and hypothalamus. Given the structure of this circuitry, alterations in the HATA could potentially affect a number of factors relevant to emotional perception and experiences including the acquisition, processing, and recall of traumatic memories, as well as contextual fear learning/conditioning^14^ and behavioral and neuroendocrine responses to traumatic stress.^85,86^ It is also thought that hippocampal and amygdala communication at a cellular level may influence cellular plasticity and further underlie contextual emotional learning and memory processing.^14^ Although these circuits are strongly implicated in PTSD,^64,87–91^ these results suggest that these memory and endocrine processing circuits may also be implicated in depression. Rather than observing the outcome of independent pathologies, it is possible that HATA subfield volume is particularly sensitive to chronic stress, a construct that overlaps with PTSD and depression psychopathology.

Depression and chronic stress are believed to increase glutamate excitotoxicity^92,93^ and glucocorticoid (GC) levels^94^ in the hippocampus, triggering inflammatory response, inhibiting neurogenesis,^95^ and lowering synaptic density.^96–98^ The present findings of reduced DG volumes are of interest, given that the DG is one of the only adult brain structures wherein neurogenesis occurs.^99^ The DG is particularly sensitive to diminished neuronal plasticity, insufficient levels of brain-derived neurotrophic factor, reduction of dendritic branching, and suppression of neurogenesis in the presence of neurotoxic GC levels.^21,96–98^ New granule cells in the DG are produced during active neurogenesis, and these cells are believed to play a key role in memory formation, pattern separation, and resolving interference between ambiguous and uncertain threat situations.^100^ In addition, the ratio of DG and CA varies as you traverse the long axis of the hippocampus, with a greater distribution of DG compared to CA subfields in the posterior aspect, which indicates that neurogenesis may be particularly relevant when discussing more posteriorly focused alterations.^101^ Given the implication of these fields in specialization across the hippocampus, it is notable that in our data set, the DG did not correlate with PTSD symptom severity, but uncorrected post hoc analyses revealed a negative association between CA2/3 and PTSD symptom severity, consistent with previous findings.^21^

Multimodal analysis of the hippocampus including, possibly, the use of histological and “brain bank” data, higher field strength MRI, and other methods may be of use in identifying what these volumetric reductions in the hippocampal subfields mean neurobiologically (i.e., reduction in cell count, projections, myelination, synaptic density, etc). More work is also necessary to understand the neurotoxic effects of stress and familial risk of PTSD^102^ and their relationship to these structural alterations; such work will be critical to properly interpret the clinical and etiological relevance of subfield volume reductions in PTSD and will help to shed additional light on the relationship between the specialization gradient across the long axis of the hippocampus^9,10^ and psychopathology.

Among the study limitations, we were unable to control for precombat childhood adversity history.^103^ Our sample, being mostly comprising male participants, cannot fully investigate potential sex differences related to PTSD or anatomy of the hippocampus, and thus may not be generalizable to female Veterans with PTSD. Given the heterogeneous nature of PTSD- and stress-related psychopathology generally, it is possible that individuals may have a slightly different mapping of structural subfield alterations. In addition, hippocampal subfields, and the HATA in particular, are relatively small regions according to the segmentation method discussed above. We have reported significant results between the HATA and symptom severity of both PTSD and depression, but it is well known that these symptoms can be highly correlated, and so multicollinearity is a concern. The reader should keep in mind that these measures are not totally independent—CAPS score explains 38% of the variance in BDI scores (*r^2^*^ ^= 0.384); however, the variance inflation factor for these measures does not suggest a strong degree of multicollinearity (VIF = 1.647). Finally, as a cross-sectional study, we did not have information about premorbid hippocampal subfield volumes. Future research should strive to elucidate whether these alterations may be a risk factor for the development of PTSD, or if they are a consequence of exposure to trauma-related pathology.

## Conclusion

In summary, this study benefits from a reasonable sample size and a dimensional approach, evaluating severity of PTSD and related symptoms, which may allow stronger inference to be made in translating volumetric findings to clinical phenotypes, including subthreshold presentations. The use of validated hippocampal subfield processing algorithms and atlases also lends strength to this study by providing a robust investigation of the hippocampal subfields based on a segmentation routine that is more reliable across subjects and equipment than previously available.^11,104^ This study makes a unique contribution to the literature by demonstrating various volumetric alterations associated with PTSD and depressive symptoms. Moreover, the results highlight the relevance of the HATA, a subfield intricately linking two regions of the brain (hippocampus and amygdala) long shown to be relevant to the study of PTSD and depression. Identification of focused relationships between symptomology and hippocampal subfields could advance our understanding of the psychopathology of trauma and chronic stress.

## Supplementary Material

Supplementary material

## Supplemental Material

The 3D hippocampal subfield video and supplemental materials are available at http://journals.sagepub.com/doi/suppl/10.1177/2470547017744538
