# Supplementary material for: Posttraumatic Stress Disorder and Depression Symptom Severities Are Differentially Associated With Hippocampal Subfield Volume Loss in Combat Veterans
Source: Chronic Stress (Thousand Oaks). 2017 Dec 13;1:2470547017744538. doi: 10.1177/2470547017744538 (PMC5839647; doi:10.1177/2470547017744538)
Supplement: Supplementary material [file supplementary_material.pdf]

Supplemental Materials:

Image Processing

FreeSurfer, which is available for download at http://surfer.nmr.mgh.harvard.edu, was used (standard recon-all pipeline) to process three structural MRI for each subject, reconstructing the cortical surface and segmenting cortical and subcortical volumes. The FreeSurfer automated pipeline is well validated and has been described in the literature. Briefly, *recon-all* automatically performed normalization of image intensity,[S1] motion correction, and averaging [S2] of two T1-weighted MPRAGE and skull stripping. [S3] Following Talairach transformation, FreeSurfer segments the gray matter (GM), subcortical white matter (WM), and deep gray matter volumetric structures. [S4, S5] The GM/WM boundary is then tessellated and overall topology is corrected. [S6, S7] To complete individual cortical modeling, the software optimizes the placement of GM/WM and GM/CSF boundaries based on shifts in intensity gradients. [S8- S10] FreeSurfer measurements have been validated against histology and manual measurements. [S10- S13] These methods use information from the entire brain volume, such as spatial intensity gradients across tissue classes. As mentioned in the manuscript, 10 GM subfields were evaluated based on enhanced subfield segmentation in FreeSurfer 6.0. [S14, S15]

Effects of Age and PTSD Symptom Severity on Subfield Volumes

Given that previous studies have reported evidence of age effect on hippocampal volumes,[S16] age was included as covariate in all primary analyses. However, to assess whether an interaction between age and PTSD severity existed, we constructed a general linear model (GLM) examining the age x PTSD symptom severity interaction. We found significant CAPS-by-age interaction in the subiculum (F=5.50, p=0.02) and the parasubiculum (F=4.71. p=0.03). No other interactive effects were found. In addition, age did not correlate with any hippocampal subfield, controlling for intracranial volume. The results of these analyses are reported below in columns 1 & 2 of Table S4.

PTSD vs Non-PTSD Group Differences

This study was optimized to conduct an analysis of PTSD symptom severity on a continuum, not to create a contrast between PTSD and non-PTSD groups. However, to inform future studies, we conducted a between group multivariate analysis removing the effect of age and estimated total intracranial volume (eTIV), and using log-transformed volumes as required for normal distribution, consistent with the methods reported in the primary manuscript. No significant differences were found between PTSD and Non-PTSD groups, but the results and estimated marginal means (by group) are reported in columns 3-5 of Table S4 below.

For completeness, we also repeated the post-hoc analyses in the Non-PTSD group, following the exploratory methods described in the manuscript for the PTSD-only group. As described in the manuscript, this post-hoc data was not corrected for multiple comparisons. No significant correlations were identified. The results are reported below in Table S5.

Table S1. Demographic and Population Details (PTSD vs Non-PTSD Subjects)

	Full Group		PTSD		Non-PTSD	
**Measure**	***N***	**Mean (SD)**	***n***	**Mean (SD)**	***n***	**Mean (SD)**
Age	68	34.78 (9.55)	36	35.03 (9.26)	32	34.50 (10.00)
WTAR IQ	65	103.06 (8.14)	33	104.12 (7.45)	32	101.97 (8.77)
Gender (Male)	61	—	32	—	29	—
Substance/Alcohol Use Disorder	13	—	10	—	3	—
Medication-Free	44	—	17	—	27	—
CES Total Score	63	18.52 (9.94)	34	21.38 (10.57)	29	15.17 (8.11)
BDI Total Score	68	18.63 (12.50)	36	25.69 (10.24)	32	10.69 (9.81)
CAPS Total Score	68	44.93 (30.75)	36	69.19 (17.23)	32	17.63 (15.96)
CAPS Reexperiencing	68	11.88 (9.33)	36	18.44 (6.73)	32	4.50 (5.55)
CAPS Arousal	68	15.68 (9.91)	36	22.94 (5.48)	32	7.50 (6.92)
CAPS Avoidance	68	5.74 (4.93)	36	8.92 (3.92)	32	2.16 (3.19)
CAPS Numbing	68	11.63 (9.82)	36	18.89 (5.98)	32	3.47 (6.16)

Abbreviations: WTAR, Wechsler Test of Adult Reading; CES, Combat Exposure Scale; BDI, Beck Depression Inventory 2nd Edition; CAPS, Clinician Administered PTSD Scale for DSM-IV.

Table S2. Demographic and Population Details (Medicated vs Non-Medicated Subjects)

	Full Group		Medicated		Non-Medicated	
**Measure**	***N***	**Mean (SD)**	***n***	**Mean (SD)**	***n***	**Mean (SD)**
Age	68	34.78 (9.55)	24	34.21 (9.79)	44	35.10 (9.51)
WTAR IQ	65	103.06 (8.14)	22	103.59 (9.67)	43	102.80 (7.34)
Gender (Male)	61	—	22	—	39	—
Substance/Alcohol Use Disorder	13	—	4	—	9	—
CES Total Score	63	18.52 (9.94)	23	21.35 (11.62)	40	16.90 (8.58)
BDI Total Score	68	18.63 (12.50)	24	26.67 (9.18)	44	14.25 (11.94)
CAPS Total Score	68	44.93 (30.75)	24	59.54 (23.82)	44	36.96 (31.39)
CAPS Reexperiencing	68	11.88 (9.33)	24	15.58 (7.88)	44	9.86 (9.53)
CAPS Arousal	68	15.68 (9.91)	24	20.46 (7.45)	44	13.07 (10.18)
CAPS Avoidance	68	5.74 (4.93)	24	7.42 (4.30)	32	4.82 (5.05)
CAPS Numbing	68	11.63 (9.82)	24	16.08 (8.71)	44	9.21 (9.62)

Abbreviations: WTAR, Wechsler Test of Adult Reading; CES, Combat Exposure Scale; BDI, Beck Depression Inventory 2nd Edition; CAPS, Clinician Administered PTSD Scale for DSM-IV. ***Full Group details are repeated from Table S1 for convenience.***

Table S3. Raw Intracranial and Bilateral Hippocampal Subfield Volumes

Volume	Full Group (*n=68)*	PTSD (*n*=36)	Non-PTSD (*n*=32)	Medicated (*n*=24)	Non-Medicated (*n*=44)
	Mean (SEM)	Mean (SEM)	Mean (SEM)	Mean (SEM)	Mean (SEM)
eTIV	1573970.59 (18360.29)	1574444.44 (25434.15)	1573437.50 (26954.30)	1561250.00 (33607.46)	1580909.09 (21887.22)
Hippocampus	8417.84 (107.01)	8301.88 (135.75)	8548.31 (167.77)	8149.11 (181.09)	8564.43 (128.80)
Subiculum	869.19 (12.44)	865.48 (16.80)	873.36 (18.74)	846.19 (21.44)	881.73 (15.10)
Presubiculum	628.10 (9.63)	631.65 (13.23)	624.11 (14.24)	609.77 (18.53)	638.09 (10.80)
Parasubiculum	128.09 (2.41)	131.37 (3.31)	124.40 (3.45)	129.41 (5.09)	127.37 (2.52)
CA1	1296.90 (17.11)	1286.51 (21.04)	1308.59 (27.81)	1254.62 (28.25)	1319.96 (20.90)
CA2/3	453.92 (7.34)	448.60 (9.93)	459.90 (10.95)	435.67 (11.39)	463.88 (9.24)
CA4	530.85 (7.23)	526.40 (8.79)	535.85 (11.85)	509.90 (10.43)	542.27 (9.25)
Dentate Gyrus	618.84 (8.54)	613.95 (10.34)	624.33 (14.04)	594.63 (12.33)	632.04 (10.94)
Molecular Layer	1169.13 (15.06)	1161.09 (18.62)	1178.18 (24.41)	1131.79 (24.82)	1189.50 (18.41)
HATA	132.17 (1.84)	129.99 (2.47)	134.63 (2.73)	128.74 (3.24)	134.04 (2.20)
Hippocampal Tail	1095.33 (16.61)	1083.32 (23.18)	1108.84 (23.94)	1053.90 (29.77)	1117.93 (19.29)

Abbreviations: CA, cornu ammonis; eTIV, estimated Total Intracranial Volume; HATA, hippocampus-amygdala transition area; Mean is volume in mm^3. SEM, Standard Error of the Mean.

Table S4. Effects of Age, CAPS Severity, and PTSD Group Status on Hippocampal Subfields

	CAPS x Age [A]	Age [B]	Group Difference [C]	PTSD	Non-PTSD
**Volume**	***F* (*p*)**	***r* (p)**	***F* (*p*)**	***EMM* (*SEM*)**	***EMM* (*SEM*)**
Subiculum	**5.50 (0.02)***	-0.18 (0.14)	0.17 (0.68)	865.61 (12.65)	873.21 (13.42)
Presubiculum	2.66 (0.11)	-0.12 (0.35)	0.28 (0.60)	631.66 (9.87)	624.10 (10.47)
Parasubiculum	**4.71 (0.03)***	-0.06 (0.62)	2.71 (0.11)	131.37 (2.91)	124.40 (3.08)
CA1	0.98 (0.33)	-0.12 (0.35)	0.90 (0.35)	1286.46 (16.02)	1308.64 (16.99)
CA2/3	0.04 (0.84)	-0.04 (0.74)	0.86 (0.36)	448.55 (8.46)	459.97 (8.98)
CA4	0.83 (0.37)	-0.12 (0.35)	0.74 (0.39)	2.72 (0.01)	2.73 (0.01)
Dentate Gyrus	1.34 (0.25)	-0.13 (0.29)	0.59 (0.45)	2.79 (0.01)	2.79 (0.01)
Molecular Layer	1.89 (0.17)	-0.20 (0.11)	0.53 (0.47)	3.06 (0.01)	3.07 (0.01)
HATA	0.64 (0.43)	0.08 (0.50)	2.55 (0.12)	129.93 (2.04)	134.69 (2.17)
Hippocampal Tail	0.58 (0.45)	-0.03 (0.82)	0.87 (0.35)	1083.15 (19.01)	1109.03 (20.16)

A) Interaction from multivariate general linear model. B) Partial correlation between subfield and age, controlling for eTIV. C) Multivariate general linear model analysis comparing PTSD and Non-PTSD groups. Abbreviations: CA, cornu ammonis; eTIV, estimated Total Intracranial Volume; HATA, hippocampus-amygdala transition area; EMM, Estimated Marginal Mean (mm3); SEM, Standard Error of the Mean. **** Effects are significant where p is less than or equal to 0.05* (p ≤ 0.05).**

Table S5. Post-hoc Correlations of PTSD Symptom Severity in Subjects Without PTSD

	**CAPS Exploratory (Non-PTSD group)**		
**Volume**	***r***	**p**	**df**
Total Hippocampus	-0.08	0.68	28
Parasubiculum	0.02	0.94	28
Presubiculum	-0.02	0.93	28
Subiculum	-0.10	0.61	28
CA1	-0.04	0.83	28
CA2/3	0.03	0.89	28
CA4	0.10	0.60	28
Dentate Gyrus	0.11	0.57	28
HATA	-0.17	0.36	28
ML of Hippocampus	-0.07	0.73	28
Hippocampal Tail	-0.16	0.39	28

Abbreviations: r, partial correlation controlling for age and total intracranial volume; CA, cornu ammonis; ML, molecular layer; DG, granule cell and molecular layers of the dentate gyrus; HATA, hippocampal-amygdala transition area; CAPS, Clinician Administered PTSD Scale for DSM-IV. Correlations were conducting controlling for the effects of age and total intracranial volume. *** indicates significance if p is less than or equal to 0.05 ($p \leq 0.05$).** Exploratory analysis—not corrected for multiple comparisons. For Full Group and PTSD-Only Group results, see Table 2 in the primary manuscript.

Video Legends:

Video S1. 10 Gray Matter Subfields of the Hippocampus – Animated 3D Model. This video was created in Blender using meshes from one of the combat control (non-PTSD) participants described in the manuscript. It demonstrates the gray matter subfields of the hippocampal formation, and steps through the anatomy to better visualize the more hidden subfields.

Supplement References

S1. Sled JG, Zijdenbos AP and Evans AC. A nonparametric method for automatic correction of intensity nonuniformity in MRI data. *IEEE Trans Med Imaging*. 1998; 17: 87-97.

S2. Reuter M, Rosas HD and Fischl B. Highly accurate inverse consistent registration: a robust approach. *Neuroimage*. 2010; 53: 1181-96.

S3. Segonne F, Dale AM, Busa E, et al. A hybrid approach to the skull stripping problem in MRI. *Neuroimage*. 2004; 22: 1060-75.

S4. Fischl B, Salat DH, Busa E, et al. Whole brain segmentation: automated labeling of neuroanatomical structures in the human brain. *Neuron*. 2002; 33: 341-55.

S5. Fischl B, Salat DH, van der Kouwe AJ, et al. Sequence-independent segmentation of magnetic resonance images. *Neuroimage*. 2004; 23 Suppl 1: S69-84.

S6. Fischl B, Liu A and Dale AM. Automated manifold surgery: constructing geometrically accurate and topologically correct models of the human cerebral cortex. *IEEE Trans Med Imaging*. 2001; 20: 70-80.

S7. Ségonne F, Pacheco J and Fischl B. Geometrically accurate topology-correction of cortical surfaces using nonseparating loops. *IEEE transactions on medical imaging*. 2007; 26: 518-29.

S8. Dale AM, Fischl B and Sereno MI. Cortical surface-based analysis. I. Segmentation and surface reconstruction. *Neuroimage*. 1999; 9: 179-94.

S9. Dale AM and Sereno MI. Improved Localizadon of Cortical Activity by Combining EEG and MEG with MRI Cortical Surface Reconstruction: A Linear Approach. *J Cogn Neurosci*. 1993; 5: 162-76.

S10. Fischl B and Dale AM. Measuring the thickness of the human cerebral cortex from magnetic resonance images. *Proc Natl Acad Sci U S A*. 2000; 97: 11050-5.

S11. Kuperberg GR, Broome MR, McGuire PK, et al. Regionally localized thinning of the cerebral cortex in schizophrenia. *Arch Gen Psychiatry*. 2003; 60: 878-88.

S12. Rosas HD, Liu AK, Hersch S, et al. Regional and progressive thinning of the cortical ribbon in Huntington's disease. *Neurology*. 2002; 58: 695-701.

S13. Salat DH, Buckner RL, Snyder AZ, et al. Thinning of the cerebral cortex in aging. *Cereb Cortex*. 2004; 14: 721-30.

S14. Iglesias JE, Augustinack JC, Nguyen K, et al. A computational atlas of the hippocampal formation using ex vivo, ultra-high resolution MRI: Application to adaptive segmentation of in vivo MRI. *Neuroimage*. 2015; 115: 117-37.

S15. Whelan CD, Hibar DP, van Velzen LS, et al. Heritability and reliability of automatically segmented human hippocampal formation subregions. *Neuroimage*. 2016; 128: 125-37.

S16. Wang Z, Neylan TC, Mueller SG, et al. Magnetic resonance imaging of hippocampal subfields in posttraumatic stress disorder. *Arch Gen Psychiatry*. 2010; 67: 296-303.
